# Emergence of *Neisseria meningitidis* W135 in Cote d’Ivoire: laboratory based-surveillance

**DOI:** 10.4178/epih.e2018058

**Published:** 2018-11-28

**Authors:** Man-Koumba Soumahoro, Clarisse Kouamé-Elogne, Jean-Claude Anné, Soualihou Noufé, Kouakou Christophe N’Guessan, Adèle Kacou-N’Douba, Thomas Hanslik, Mireille Dosso

**Affiliations:** 1 Département Epidémiologie Recherche Clinique, Institut Pasteur de Côte d’Ivoire, Abidjan, Cote d’lvoire; 2 Département Bactériologie Virologie, Institut Pasteur de Côte d’Ivoire, Abidjan, Cote d’lvoire; 3 Institut National de l’Hygiène Publique, Abidjan, Cote d’lvoire; 4 Institut National de la Santé Publique, Abidjan, Cote d’lvoire; 5 Université Félix Houphouët Boigny, Unité de Formation et de Recherche Sciences Médicales, Abidjan, Cote d’lvoire; 6 Université de Versailles-Saint-Quentin, Montigny-le-Bretonneux, France; 7 Assistance Publique-Hôpitaux de Paris, Hôpital Ambroise Paré, Service de Médecine Interne, Boulogne Billancourt, France

**Keywords:** Bacterial meningitis, *Neisseria meningitidis*, Public health surveillance, Cote d’Ivoire

## Abstract

**OBJECTIVES:**

To describe the emergence of *Neisseria meningitidis* (Nm) W135 in Côte d’Ivoire and its characteristics compared to NmA.

**METHODS:**

Data on Nm samples isolated at the National Reference Center for meningitis in Côte d’Ivoire between 2007 and 2012 were analyzed. Socio-demographic data and biological information on the samples were extracted from the database. Categorical variables, such as sex and the serotype of the bacteria, were compared using the Fisher exact test, while the distribution of continuous variables, such as age, was compared using the Wilcoxon test.

**RESULTS:**

Among the 175 Nm samples, 57 were NmA, 4 were NmB, 13 were NmC, and 99 were NmW135. The geographical distribution of NmA and NmW135 did not show a significant difference according to age or sex. NmW135 was more common than NmA in the northern health districts of Cote d’Ivoire (85.9 vs. 45.5%; p<0.001). No sample of NmA has been isolated since 2009, while 95% of the type W135 samples were isolated between 2010 and 2012.

**CONCLUSIONS:**

This study highlighted the emergence of NmW135 in Côte d’Ivoire, as well as the simultaneous disappearance of NmA. It is important to improve laboratory-based surveillance of meningitis to assess trends in the circulation of bacteria and to detect the emergence of new serogroups earlier.

## INTRODUCTION

Meningitis is an infection of the membranes (meninges) surrounding the brain and spinal cord. It is associated with a high case fatality rate (up to 50% when not treated) and a high frequency of severe sequelae (more than 10%) [1]. Bacterial meningitis is a major public health concern in Côte d’Ivoire (CI). Meningococci are exclusively pathogenic in humans and are transmitted directly from person to person by droplets of respiratory or pharyngeal secretions. Meningococcal disease is the leading cause of bacterial meningitis in sub-Saharan Africa [2,3]. Seasonal epidemics due to *Neisseria meningitidis* (Nm) occur during the dry season between December and June, and they are most often localized [2].

In 2000 and 2001, epidemics of meningitis occurred in Saudi Arabia, during which more than half of cases were confirmed as NmW135 [4,5]. Since then, several imported cases have been reported around the world [6-8]. In 2001, serogroup W135–related meningitis epidemics in Burkina Faso and Niger resulted in 13,039 and 7,906 cases, respectively [9,10]. Then, in 2002, outbreaks due to serogroup W135 were observed in Burkina Faso, while a few imported cases were reported in Senegal and CI [11-13].

In 2012, NmW135, which had previously sporadically circulated in CI, caused outbreaks in the health districts of Tengrela and Korhogo, located in northern CI [14]. The objective of this study was to describe the emergence of NmW135 in CI, as well as its epidemiological characteristics compared to those of NmA.

## MATERIALS AND METHODS

The National Reference Center of meningitis (NRCm) of CI receives cerebrospinal fluid (CSF) specimens from suspected meningitis cases, collected by doctors from the country’s health facilities, for bacteriological confirmation as part of the national integrated surveillance and response program for the disease. Samples are sent to the Institut Pasteur of CI, which houses the NRCm, through the National Institute of Public Hygiene. All suspected cases cannot be systematically collected. However, the NRCm receives samples from on average nearly 20% of suspected cases of meningitis. During outbreaks, these 2 institutions often jointly organize sample collection campaigns in various health districts.

The biological diagnosis of bacterial meningitis was based on a combination of tests, including culture, the latex agglutination test, and polymerase chain reaction. A case of meningococcal meningitis is defined by the presence of Nm in the CSF.

Data from the NRCm in CI were analyzed between 2007 and 2012. These data included information on the socio-demographic characteristics of the patients whose CSF had been sampled (age, sex, health district) and the biological characteristics of the samples (date of arrival at the NRCm, macroscopic appearance, pleocytosis, culture, isolated bacteria, meningococcal serogroup). All these data were collected from standardized report forms. Data were entered using Epidata version 2.0 and analyzed with Stata version 11 (StataCorp., College Station, TX, USA). Categorical variables were tabulated using frequencies and proportions, and they were compared using the Fisher exact test. For continuous variables, results were summarized as medians and interquartile ranges (IQR). The ages of confirmed cases of NmW135 and NmA were compared using the Wilcoxon test. The analysis utilized an alpha significance level of 0.05, with 2-sided tests.

## RESULTS

Between 2007 and 2012, 4637 CSF samples were received at NRCm. Most samples were from people living in the Abidjan-2 health region (3,954 of 4,637; 85.3%), which is located in the forest zone in the south of CI. More than half of the samples were from children under 5 years of age (2,543 of 4,460; 57.0%). The median age was 4 years (IQR, 1 to 15 years) and the male-to-female ratio was 0.87.

The bacteriological analysis revealed bacterial meningitis in 8.7% of cases (405 of 4,637). These cases were caused by Nm, *Streptococcus pneumoniae, Haemophilus influenza* type b, and group B Streptococcus in 175 (43.2%), 158 (39.0%), 58 (14.3%) and 14 (3.5%) cases, respectively (Table 1).

Among the 175 Nm isolates from 2007 to 2012, serogroup W135 accounted for 56.6% and serogroups A, C, and B for 32.6, 7.4, and 2.3%, respectively.

**Figure 1.**
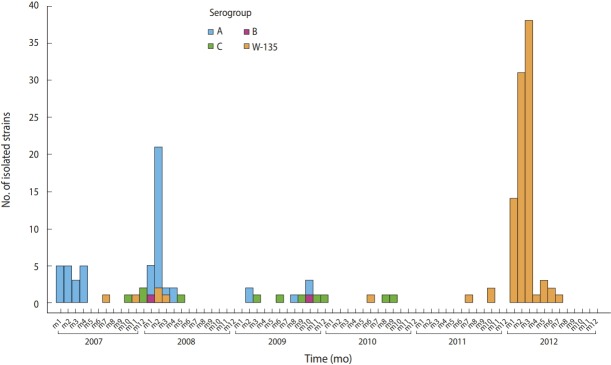
Trends in the frequency of meningococcal strains isolated at the National Reference Center for meningitis from 2007 to 2012 by serogroup, Cote d’Ivoire.

NmA was not isolated between 2010 and 2012, while 95% of W135 samples were isolated during this period (Figure 1).

**Figure 2.**
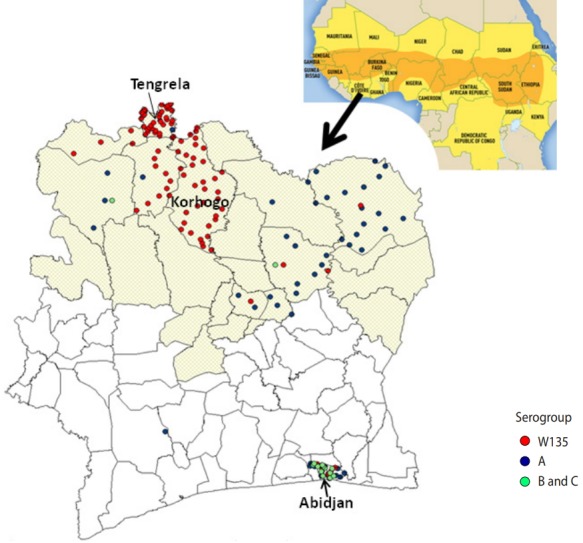
Spatial distribution of meningococcus strains isolated at the National Reference Center for meningitis from 2007 to 2012 by serogroup, Cote d’Ivoire.

Most of the meningococcal samples (112; 64.7%), were from patients living in the health districts located in the meningitis belt, in northern CI (Table 1 and Figure 2)

Meningitis was most often found in patients living in the health districts of Tengrela (22.0%) and Korhogo (20.2%), located in the northern part of the country.

The median age (IQR) of the confirmed cases of meningococcal disease was 9 (7 to 11) years. The male-to-female ratio of meningococcal cases was 1.4. The median age of patients with the meningococcal serogroups A and W135 was 12 years (5 to 16) and 7 years (3 to 16), respectively (p=0.20). An analysis of the age distributions in these 2 groups showed no significant differences. Likewise, no significant relationship was found between serogroup and sex (p=0.60). NmW135 was more often isolated than NmA in CSF samples from health districts in the meningitis belt (85.9 vs. 45.5%; p<0.001).

## DISCUSSION

This study documented the emergence of serogroup W135 in CI. In fact, 95% of NmW135 samples were isolated between 2010 and 2012, while NmA has not been isolated since 2009. Before the 2012 outbreaks, serogroup W135 sporadically circulated and epidemics were mainly serogroup A–related [13]. The latter serogroup was responsible for epidemics between 2004 and 2008 in 6 health districts in the northern part of the country. Between 2007 and 2012, 2,951 cases of cerebrospinal meningitis with a case-fatality rate of 17.9% were reported to the World Health Organization (source: National Institute of Public Hygiene).

The African meningitis belt, described in 1963 by Lapeyssonnie [15], extends from Senegal in the west to Ethiopia in the east of Africa. Meningococcal disease affects this area of sub-Saharan Africa more heavily, and this area encompasses 26 countries (Figure 2). CI is bordered to the north by Mali and Burkina Faso. The north of CI is located in the southern boundary of the African meningitis belt, and 7 regions in the north of CI have been identified as at risk for outbreaks of meningitis (Figure 2).

In sub-Saharan Africa, the emergence of NmW135 in countries located in the meningitis belt followed epidemics that occurred in 2000-2001. Thus, in 2002, Burkina Faso faced its first large-scale epidemic due to NmW135, which caused nearly 1,510 deaths out of 13,124 reported cases, with an average lethality of 11.5% [16]. Few cases of meningitis due to NmW135 were recorded between 2000 and 2009 in Senegal [12]. The movement of people undoubtedly favored the emergence of this serogroup in sub-Saharan Africa. Analysis of the NRCm data revealed that the epidemic foci of meningococcal disease related to NmA and NmW135 were observed in health districts close to Mali and Burkina Faso, respectively. In addition, the introduction of a monovalent conjugate vaccine against through large vaccination campaigns in some countries such as Burkina Faso, Mali, and Niger may have contributed to the decline of NmA and thereby favored the emergence of Nm W135, although this hypothesis could not be investigated in this study [3,17].

The various vaccination campaigns carried out before 2012 were followed by a sharp decrease in the circulation of NmA. Indeed, several districts in the at-risk area received large-scale vaccinations against NmA. Because the population remained largely susceptible to NmW135, this could explain the regional difference between serotypes. With the emergence of NmW135 and other serogroups (X, Y), the bivalent polysaccharide vaccine against serogroup A and C faces limits, leading to interest in promoting the tetravalent conjugate vaccine or the pentavalent conjugate vaccine.

There is no clinical difference depending on the causative microorganism. The relevant public health issue relates to vaccination strategy. Until 2012, epidemics caused by Nm were mainly due to serotype A. The trend was to immunize the population against this serotype. After the emergence of NmW135, it has become important to adapt the preventive strategies that have been put into place and to choose vaccines directed against the type of meningitis circulating in the country. This, in turn, suggests the need to strengthen the microbiological surveillance of micro-organisms that cause bacterial meningitis.

Finally, this study highlighted the emergence of NmW135 in CI, as well as the simultaneous disappearance of NmA. Of further note, some cases were found in health districts in the southern part of the country (i.e., not in the meningitis belt). It is important to improve the laboratory-based surveillance of Nm to assess trends in bacterial circulation and to detect the emergence of new serogroups earlier. Conducting surveys of *Meningococcus* transmission during inter-epidemic periods could inform vaccination strategies against meningitis epidemics linked to Nm in CI.

## Figures and Tables

**Table 1.** Characteristics of cases of confirmed bacterial meningitis and meningococcal disease at the National Reference Center for meningitis 2007-2012, Cote d’Ivoire

Characteristics	Bacterial meningitis (n=405)	Meningococcal (n=175)	Serogroup		
			A (n=57)	W135 (n=99)	p-value^1^
Demographic					
Sex (female)	192/397 (48.4)	73/172 (42.4)	22 (38.6)	42/96 (43.8)	0.60
Age, median [IQR]	5 [1-14]	9 [7-11]	12 [5-16]	7 [3-16]	0.20^2^
Located in meningitis belt (yes)	143/398 (35.9)	112/173 (64.7)	25/55 (45.5)	85/99 (85.9)	<0.001
Case confirmation period					
2007-2009	180 (44.4)	77 (44.0)	57 (100)	5 (5.0)	<0.001
2010-2012	225 (55.6)	98 (56.0)	0 (0.0)	94 (95.0)	
Microbiological					
Abnormal/purulent CSF	201/398 (50.5)	87/170 (51.2)	30 (52.6)	43/94 (45.7)	0.50
Pleocytosis (yes)	253/398 (63.6)	94/171 (55.0)	37 (64.9)	42/95 (44.2)	0.01
Positive culture	175/404 (43.3)	62/174 (35.6)	21 (36.8)	30/98 (30.6)	0.50
Bacteria isolated					
*Neisseria meningitis* (serogroup)	175 (43.2)	-	-	-	-
W135	99 (56.6)	-	-	-	-
A	57 (32.6)	-	-	-	-
C	13 (7.4)	-	-	-	-
B	4 (2.3)	-	-	-	-
Unknown	2 (1.1)	-	-	-	-
*Streptococcus pneumoniae*	158 (39.0)	-	-	-	-
*Haemophilus influenzae* type b	58 (14.3)	-	-	-	-
Group B *Streptococcus*	14 (3.5)	-	-	-	-

Values are presented as number (%). IQR, interquartile range; CSF, cerebrospinal fluid.

1 Fisher exact test.

2 Wilcoxon test for median comparison.
